# Escherichia coli Nissle 1917 Enhances Innate and Adaptive Immune Responses in a Ciprofloxacin-Treated Defined-Microbiota Piglet Model of Human Rotavirus Infection

**DOI:** 10.1128/mSphere.00074-21

**Published:** 2021-03-31

**Authors:** Husheem Michael, Francine C. Paim, Stephanie N. Langel, Ayako Miyazaki, David D. Fischer, Juliet Chepngeno, Joshua Amimo, Loic Deblais, Gireesh Rajashekara, Linda J. Saif, Anastasia N. Vlasova

**Affiliations:** a Food Animal Health Research Program, Department of Veterinary Preventive Medicine, Ohio Agricultural Research and Development Center, The Ohio State University, Wooster, Ohio, USA; b Division of Viral Disease and Epidemiology, National Institute of Animal Health, National Agriculture and Food Research Organization, Tsukuba, Ibaraki, Japan; University of Michigan—Ann Arbor

**Keywords:** probiotics, human rotavirus infection, innate immunity, adaptive immunity, ciprofloxacin, gnotobiotic pigs, commensal microbiota

## Abstract

Human rotavirus (HRV) infection is a major cause of gastroenteritis in children worldwide. Broad-spectrum antibiotic-induced intestinal microbial imbalance and the ensuing immune-metabolic dysregulation contribute to the persistence of HRV diarrhea. Escherichia coli Nissle 1917 (EcN), a Gram-negative probiotic, was shown to be a potent immunostimulant and alleviated HRV-induced diarrhea in monocolonized gnotobiotic (Gn) piglets. Our goal was to determine how EcN modulates immune responses in ciprofloxacin (Cipro)-treated Gn piglets colonized with a defined commensal microbiota (DM) and challenged with virulent HRV (VirHRV). Cipro given in therapeutic doses for a short term reduced serum and intestinal total and HRV-specific antibody titers, while EcN treatment alleviated this effect. Similarly, EcN treatment increased the numbers of total immunoglobulin-secreting cells, HRV-specific antibody-secreting cells, activated antibody-forming cells, resting/memory antibody-forming B cells, and naive antibody-forming B cells in systemic and/or intestinal tissues. Decreased levels of proinflammatory but increased levels of immunoregulatory cytokines and increased frequencies of Toll-like receptor-expressing cells were evident in the EcN-treated VirHRV-challenged group. Moreover, EcN treatment increased the frequencies of T helper and T cytotoxic cells in systemic and/or intestinal tissues pre-VirHRV challenge and the frequencies of T helper cells, T cytotoxic cells, effector T cells, and T regulatory cells in systemic and/or intestinal tissues postchallenge. Moreover, EcN treatment increased the frequencies of systemic and mucosal conventional and plasmacytoid dendritic cells, respectively, and the frequencies of systemic natural killer cells. Our findings demonstrated that Cipro use altered immune responses of DM-colonized neonatal Gn pigs, while EcN supplementation rescued these immune parameters partially or completely.

**IMPORTANCE** Rotavirus (RV) is a primary cause of malabsorptive diarrhea in children and is associated with significant morbidity and mortality, especially in developing countries. The use of antibiotics exacerbates intestinal microbial imbalance and results in the persistence of RV-induced diarrhea. Probiotics are now being used to treat enteric infections and ulcerative colitis. We showed previously that probiotics partially protected gnotobiotic (Gn) piglets against human RV (HRV) infection and decreased the severity of diarrhea by modulating immune responses. However, the interactions between antibiotic and probiotic treatments and HRV infection in the context of an established gut microbiota are poorly understood. In this study, we developed a Gn pig model to study antibiotic-probiotic-HRV interactions in the context of a defined commensal microbiota (DM) that mimics aspects of the infant gut microbiota. Our results provide valuable information that will contribute to the treatment of antibiotic- and/or HRV-induced diarrhea and may be applicable to other enteric infections in children.

## INTRODUCTION

Human rotavirus (HRV) is a leading cause of malabsorptive diarrhea in children and causes significant morbidity and mortality, especially in developing countries (1–3). The frequent use of antibiotics exacerbates intestinal microbial imbalance and often correlates with the persistence of HRV-induced diarrhea (4). Therefore, alternative strategies are needed to ameliorate infectious viral diarrhea.

Probiotics have been shown to enhance immune responses to oral vaccines (5, 6) and have been used to treat enteric infections (7) and ulcerative colitis (8) in children. Furthermore, they inhibit Helicobacter pylori growth (9), prevent cancer (10–12), decrease gut inflammation (13), and prevent allergies (14, 15). The Gram-negative probiotic Escherichia coli Nissle 1917 (EcN) has been widely used in the treatment of ulcerative colitis in humans (16). EcN can become established in the gut microbiome (17). We have shown previously that EcN partially protected gnotobiotic (Gn) piglets against HRV infection and decreased the severity of diarrhea by modulating innate and adaptive immunity and protecting the intestinal epithelium by binding HRV particles via histo-blood group antigen-like bacterial glycans (18–20).

Gn pigs are immunocompetent at birth but immunologically immature (21). HRV-infected Gn pigs exhibit diarrhea, transient viremia, and intestinal lesions mimicking natural human rotavirus infection in children (22, 23). Gn pigs are caesarian derived and housed in sterile isolators to ensure their germfree status, permitting studies of gut colonization with single bacteria or a defined or fecal microbiota. Thus, Gn pigs are a unique model to study host metabolism, neonatal immune responses, enteric viral infections, or oral vaccines without confounding the microbiota (24, 25). Although Gn pig models have been used to study the effects of vaccines and probiotic treatments in HRV-challenged pigs, studies examining the interactions between these treatments and HRV infection in the context of the microbiota are limited. We developed a simplified model that mimics the infant gut microbiota by transplanting Gn pigs with a defined commensal microbiota (DM) (20, 26). The DM, with a composition similar to that of the modified Schaedler flora used in mice, consists of seven bacterial species of swine origin (Bifidobacterium adolescentis, Bifidobacterium longum, Bacteroides thetaiotaomicron, Enterococcus faecalis, Lactobacillus brevis, Streptococcus bovis, and Clostridium clostridioforme) (27).

These bacterial species are predominant in neonates (28–30). Hence, our DM-Gn pig model mimics major infant gut microbiota for investigating natural human HRV infection and the interactions between antibiotics, probiotics, and intestinal commensals (20, 26). Our previous study focused on the use of DM-transplanted Gn pigs treated concurrently with ciprofloxacin (Cipro) and EcN and infected with HRV. EcN treatment affected the intestinal epithelium by increasing the gene expression of enteroendocrine and enterocyte cells, maintaining the absorptive function, and thus ameliorating HRV diarrhea severity aggravated by Cipro treatment (*P ≤ *0.05) (20). Moreover, EcN treatment enhanced the bacterial diversity of all seven DM species and alleviated the adverse impacts of Cipro treatment during acute HRV diarrhea (26). In addition to the protection of the gut epithelium and microbiota modulation, attenuation of HRV diarrhea severity may be associated with EcN-mediated intestinal and systemic immune responses.

The purpose of this study was to investigate the effects of EcN with Cipro on the immune responses to HRV in treated DM-transplanted Gn pigs. Our findings demonstrate that EcN treatment enhanced adaptive and innate immune responses. Our results emphasize that the DM-Gn pig is a suitable and robust model to study human enteric viral infections and the effects of various therapies such as probiotics and antibiotics.

## RESULTS

### EcN treatment increased the numbers of HRV-specific IgA antibody-secreting cells in systemic and intestinal tissues and increased the HRV-specific IgA antibody titers in serum, small intestinal contents, and large intestinal contents in Cipro-treated DM pigs after VirHRV challenge.

**FIG 1:**
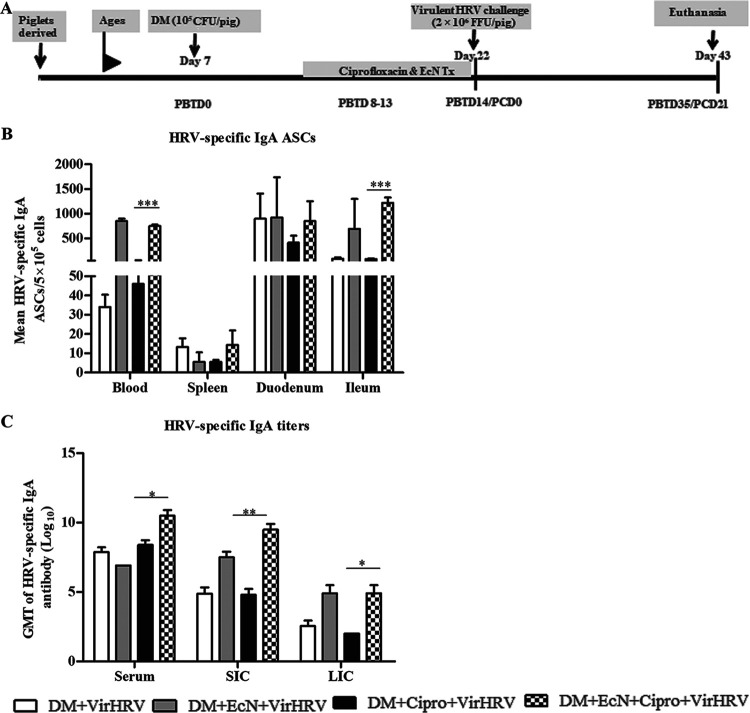
Escherichia coli Nissle 1917 (EcN) treatment enhanced HRV-specific IgA antibody-secreting cells (ASCs) and HRV-specific IgA antibody titers in defined commensal microbiota (DM)-transplanted pigs with or without ciprofloxacin (Cipro) after virulent human rotavirus (VirHRV) challenge. (A) Schematic diagram of the experimental design showing the time points for DM transplantation, EcN and Cipro treatment (Tx), VirHRV challenge, and euthanasia. (B) Mean numbers of HRV-specific IgA ASCs in systemic and intestinal tissues. (C) Geometric mean titers (GMT) of HRV-specific IgA antibodies in serum, small intestinal contents (SIC), and large intestinal contents (LIC). Data are shown as means ± SEM for the EcN/Cipro versus the Cipro groups. Significant differences are indicated (*, *P < *0.05; **, *P < *0.01; ***, *P < *0.001), as calculated from a nonparametric Kruskal-Wallis rank sum test. Gnotobiotic (Gn) neonatal piglets were derived using hysterectomy and transplanted with DM at 7 days of age, followed by challenge with VirHRV 14 days later, and pigs were euthanized at 3 weeks postchallenge (postchallenge day 21 [PCD21]). PBTD, post-bacterial transplantation day.

EcN with or without Cipro treatment following virulent HRV (VirHRV) challenge was investigated. EcN treatment concurrent with Cipro (EcN+Cipro) increased the mean numbers of HRV-specific immunoglobulin A (IgA) antibody-secreting cells (ASCs) in blood and ileal tissues (Fig. 1B). Diarrheal scores and HRV fecal shedding were recorded daily after VirHRV challenge for up to 7 days, and it was reported previously that EcN treatment ameliorated HRV diarrheal severity (see Fig. S1 in the supplemental material) (20). HRV-specific IgA ASCs in the blood, spleen, duodenum, and ileum were negatively correlated with diarrheal scores (*R* = −0.4 [*P *= 0.05], *R* = −0.4 [*P* = 0.05], *R* = −0.5 [*P *= 0.02], and *R* = −0.6 [*P *= 0.008], respectively).

10.1128/mSphere.00074-21.1 FIG S1 Escherichia coli Nissle 1917 (EcN) treatment induces protection against HRV-induced viral shedding and diarrhea in ciprofloxacin (Cipro)-treated piglets. (A) Viral shedding determined by cell culture immunofluorescence assays and expressed as FFU per milliliter. (B) Duration of diarrhea was determined by the number of days with a fecal score of >1 (fecal consistency was scored as 0 for normal, 1 for pasty/semiliquid, and 2 for liquid). (C) Mean cumulative fecal scores (daily fecal scores from postchallenge day 1 [PCD1] to PCD7) to assess the severity and percentage of diarrhea. Significant differences were calculated by a nonparametric Kruskal-Wallis rank sum test. Gnotobiotic (Gn) neonatal piglets were derived using hysterectomy and transplanted with defined commensal microbiota (DM) at 7 days of age, followed by challenge with virulent human rotavirus (VirHRV) 14 days later, and pigs were euthanized at 3 weeks postchallenge (PCD21). PBTD, post-bacterial transplantation day. Download FIG S1, TIF file, 0.03 MB. Copyright © 2021 Michael et al. 2021 Michael et al. https://creativecommons.org/licenses/by/4.0/ This content is distributed under the terms of the Creative Commons Attribution 4.0 International license.

IgA ASCs in the duodenum were negatively correlated with VirHRV shedding (*R* = −0.5 [*P *= 0.02]). EcN treatment increased the HRV-specific IgA antibody titers in serum, small intestinal contents (SIC), and large intestinal contents (LIC) (Fig. 1C). Moreover, HRV-specific IgA titers in serum, SIC, and LIC were negatively correlated with diarrheal scores (*R* = −0.4 [*P *= 0.05], *R* = −0.7 [*P *= 0.0007], and *R* = −0.5 [*P *= 0.03], respectively).

Similar trends were observed for HRV-specific IgG and IgM ASC numbers in systemic and intestinal tissues and HRV-specific IgG and IgM antibody titers in serum, SIC, and LIC (Fig. S2). HRV-specific IgM ASC numbers were below the detection limit in blood tissues. HRV-specific IgG titers in LIC were negatively correlated with the diarrheal score (*R* = −0.6 [*P* = 0.008]). A similar trend was also observed in total Ig-secreting cells (IgSCs) and total Ig isotype concentrations (Fig. S3). Total IgM IgSCs in the spleen and ileum were negatively correlated with the diarrhea score (*R* = −0.6 [*P* = 0.004] and *R* = −0.6 [*P* = 0.004], respectively). IgA IgSCs in blood were negatively correlated with VirHRV shedding (*R* = −0.5 [*P* = 0.02]).

10.1128/mSphere.00074-21.2 FIG S2 Escherichia coli Nissle 1917 (EcN) treatment modulated HRV-specific IgG and IgM antibody-secreting cells (ASCs) and HRV-specific IgG and IgM antibody titers in defined commensal microbiota (DM)-transplanted pigs with or without ciprofloxacin (Cipro) after virulent human rotavirus (VirHRV) challenge. (A and B) Mean numbers of HRV-specific IgG (B) and IgM (B) ASCs in systemic and intestinal tissues. (C and D) Geometric mean titers (GMT) of HRV-specific IgG (C) and HRV-specific IgM (D) antibody titers in serum, small intestinal contents (SIC), and large intestinal contents (LIC). Data are shown as means ± SEM for the EcN/Cipro versus the Cipro groups. Significant differences are indicated (**, *P < *0.01), as calculated by a nonparametric Kruskal-Wallis rank sum test. Gnotobiotic (Gn) neonatal piglets were derived using hysterectomy and transplanted with DM at 7 days of age, followed by challenge with VirHRV 14 days later, and pigs were euthanized at 3 weeks postchallenge (postchallenge day 21 [PCD21]). PBTD, post-bacterial transplantation day. Download FIG S2, TIF file, 0.07 MB. Copyright © 2021 Michael et al. 2021 Michael et al. https://creativecommons.org/licenses/by/4.0/ This content is distributed under the terms of the Creative Commons Attribution 4.0 International license.

10.1128/mSphere.00074-21.3 FIG S3 Escherichia coli Nissle 1917 (EcN) treatment modulated total immunoglobulin-secreting cells (IgSCs) and total Ig concentrations in defined commensal microbiota (DM)-transplanted pigs with or without ciprofloxacin (Cipro) after virulent human rotavirus (VirHRV) challenge. (A to C) Mean numbers of total IgA IgSCs (A), total IgG IgSCs (B), and total IgM IgSCs (C) in systemic and intestinal tissues. (D to F) Total IgA concentrations (D), total IgG concentrations (E), and total IgM concentrations (F) in serum, small intestinal contents (SIC), and large intestinal contents (LIC). Data are shown as means ± SEM for the EcN/Cipro versus the Cipro groups. Significant differences are indicated (***, *P < *0.001) and were obtained from a nonparametric Kruskal-Wallis rank sum test. Gnotobiotic (Gn) neonatal piglets were derived using hysterectomy and transplanted with DM at 7 days of age, followed by challenge with VirHRV 14 days later, and pigs were euthanized at 3 weeks postchallenge (postchallenge day 21 [PCD21]). PBTD, post-bacterial transplantation day. Download FIG S3, TIF file, 0.09 MB. Copyright © 2021 Michael et al. 2021 Michael et al. https://creativecommons.org/licenses/by/4.0/ This content is distributed under the terms of the Creative Commons Attribution 4.0 International license.

### EcN+Cipro treatment increased the frequencies of activated antibody-forming B cells, Ig-secreting B cells, and resting/memory antibody-forming B cells in systemic and intestinal tissues.

**FIG 2:**
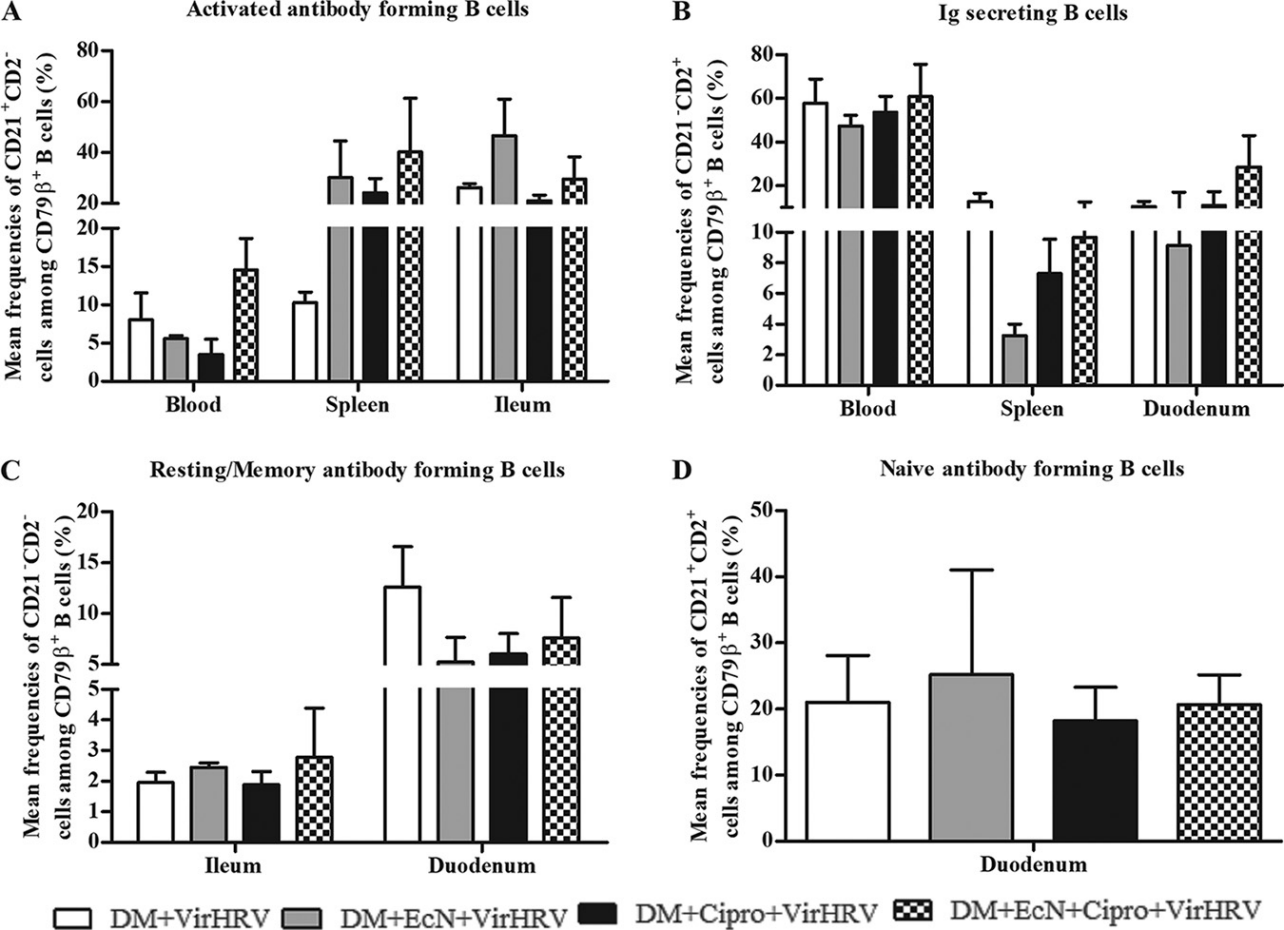
Escherichia coli Nissle 1917 (EcN) treatment alters the frequencies of antibody- and Ig-forming B cells in systemic and intestinal tissues in defined commensal microbiota (DM)-transplanted pigs with or without ciprofloxacin (Cipro) after virulent human rotavirus (VirHRV) challenge. (A) Mean frequencies of activated antibody-forming B cells (CD79β^+^ CD2^+^ CD21^−^) in systemic and ileal tissues. (B) Mean frequencies of Ig-secreting B cells (CD79β^+^ CD2^−^ CD21^+^) in systemic and duodenal tissues. (C) Mean frequencies of resting/memory antibody-forming B cells (CD79β^+^ CD2^−^ CD21^−^) in intestinal tissues. (D) Mean frequencies of naive antibody-forming B cells (CD79β^+^ CD2^+^ CD21^+^) in duodenal tissues. Data are shown as means ± SEM. Statistical significance was determined by the nonparametric Kruskal-Wallis test for the EcN/Cipro versus the Cipro groups. Gn neonatal piglets were derived using hysterectomy and transplanted with DM at 7 days of age, followed by challenge with VirHRV 14 days later, and pigs were euthanized at 3 weeks postchallenge (PCD21).

Coincident with increased HRV-specific ASCs and antibody titers and reduced diarrheal scores, EcN-treated pigs had increased frequencies of CD79β^+^ CD2^+^ CD21^−^ cells in systemic and ileal tissues (Fig. 2A; Fig. S4), while no differences were observed in duodenal tissues (data not shown). The frequencies of activated antibody-forming B cells in the blood and ileum were negatively correlated with diarrheal scores (*R* = −0.4 [*P* = 0.05] and *R* = −0.5 [*P* = 0.02], respectively). Similarly, EcN treatment increased the frequencies of Ig-secreting cells in systemic and duodenal tissues (Fig. 2B; Fig. S4), while no differences were observed in ileal tissues (data not shown). EcN treatment increased the frequencies of resting/memory antibody-forming B cells in intestinal tissues (Fig. 2C; Fig. S4), but no differences were observed in systemic tissues (data not shown). Resting/memory antibody-forming B cells in the ileum were negatively correlated with diarrheal scores (*R* = −0.4 [*P* = 0.04]). Finally, EcN treatment marginally increased the frequencies of naive antibody-forming B cells in duodenal tissues only (Fig. 2D; Fig. S4), but no differences were observed in other tissues (data not shown).

10.1128/mSphere.00074-21.4 FIG S4 Escherichia coli Nissle 1917 (EcN) treatment modulated the frequencies of antibody- and Ig-forming B cells in systemic and intestinal tissues in defined commensal microbiota (DM)-transplanted pigs with or without ciprofloxacin (Cipro) after virulent human rotavirus (VirHRV) challenge. Mean frequencies of antibody-forming B cells among CD79β^+^ B cells in various tissues are shown. All piglets were derived using hysterectomy and transplanted with DM at 7 days of age, followed by challenge with VirHRV 14 days later, and pigs were euthanized at 3 weeks postchallenge (postchallenge day 21 [PCD21]). PBTD, post-bacterial transplantation day. Download FIG S4, TIF file, 0.3 MB. Copyright © 2021 Michael et al. 2021 Michael et al. https://creativecommons.org/licenses/by/4.0/ This content is distributed under the terms of the Creative Commons Attribution 4.0 International license.

### EcN treatment decreased T helper cell frequencies prechallenge but increased them postchallenge (blood and duodenum), and T cytotoxic cell frequencies were mainly increased (except in blood) pre-/post-VirHRV challenge.

**FIG 3:**
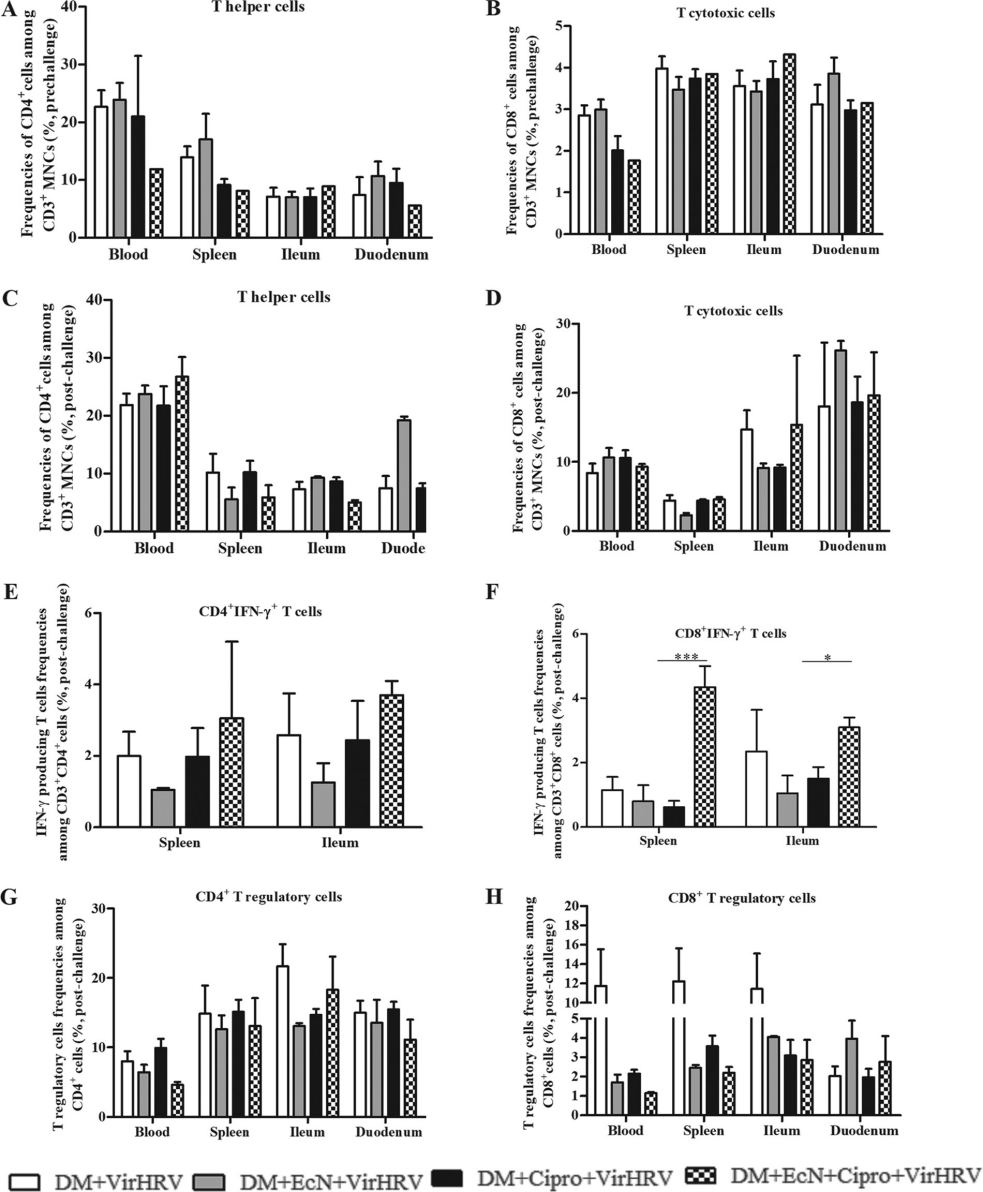
Escherichia coli Nissle 1917 (EcN) treatment alters the frequencies of T helper cells, T cytotoxic cells, HRV-specific IFN-γ-producing T cells, and T regulatory cells in systemic and intestinal tissues from defined commensal microbiota (DM)-transplanted pigs with or without ciprofloxacin (Cipro) before/after virulent human rotavirus (VirHRV) challenge. (A to D) Mean frequencies of T helper cells (CD3^+^ CD4^+^) (A) and T cytotoxic cells (CD3^+^ CD8^+^) (B) prechallenge and T helper cells (C) and T cytotoxic cells (D) postchallenge. (E and F) Mean frequencies of HRV-specific CD3^+^ CD4^+^ IFN-γ-producing T cells (E) and HRV-specific CD3^+^ CD8^+^ IFN-γ-producing T cells (F) postchallenge. (G and H) Mean frequencies of CD4^+^ CD25^+^ FOXP3^+^ T regulatory cells (G) and CD8^+^ CD25^+^ FOXP3^+^ T regulatory cells (H) postchallenge. Data are shown as means ± SEM for the EcN/Cipro versus the Cipro groups, and significant differences are indicated (*, *P < *0.05; ***, *P < *0.001), as calculated by a nonparametric Kruskal-Wallis rank sum test. Gn neonatal piglets were derived using hysterectomy and transplanted with DM at 7 days of age, followed by challenge with VirHRV 14 days later, and pigs were euthanized at 3 weeks postchallenge (PCD21).

EcN treatment decreased the frequencies of T helper cells (CD3^+^ CD4^+^) in the ileum (Fig. 3A and C). The frequencies of T cytotoxic cells (CD3^+^ CD8^+^) in the spleen and intestinal tissues were increased pre-/post-VirHRV challenge (Fig. 3B and D). The frequencies of T helper cells in blood and spleen were negatively correlated with VirHRV shedding (*R* = −0.5 [*P *= 0.003] and *R* = −0.5 [*P *= 0.03], respectively) and diarrheal scores (*R* = −0.7 [*P *= 0.006]). A similar trend was observed for T cytotoxic cells in blood, which were negatively correlated with VirHRV shedding (*R* = −0.5 [*P *= 0.04]).

### EcN+Cipro treatment increased HRV-specific IFN-γ-producing CD4 and CD8 T cell frequencies in spleen and ileum post-VirHRV challenge.

EcN treatment increased the frequency of HRV-specific CD3^+^ CD4^+^ interferon gamma (IFN-γ)-producing T cells post-VirHRV challenge in splenic and ileal tissues (Fig. 3E). Furthermore, EcN treatment increased the frequency of HRV-specific CD3^+^ CD8^+^ IFN-γ-producing T cells in splenic and ileal tissues (Fig. 3F). We observed that CD3^+^ CD8^+^ IFN-γ T cells in the spleen and ileum were negatively correlated with diarrheal scores (*R* = −0.5 [*P* = 0.03] and *R* = −0.4 [*P* = 0.05], respectively).

### EcN with or without Cipro treatment reduced T regulatory cell frequencies after VirHRV challenge.

EcN with or without Cipro treatment reduced the frequencies of CD4^+^ CD25^+^ FOXP3^+^ T regulatory cells (Tregs) and CD8^+^ CD25^+^ FOXP3^+^ Tregs post-VirHRV challenge in the blood, spleen, and ileal tissues (except CD4) and in the duodenal tissues (except CD8), respectively (Fig. 3G and H). Moreover, CD4^+^ Tregs in ileal tissues were negatively correlated with diarrheal scores (*R* = −0.5 [*P *= 0.05]). Similarly, CD4^+^ Tregs in the ileum, blood, and spleen were negatively correlated with VirHRV shedding (*R* = −0.5 [*P* = 0.05], *R* = −0.7 [*P *= 0.02], and *R* = −0.6 [*P *= 0.02], respectively).

### EcN with or without Cipro treatment reduced proinflammatory and increased immunoregulatory cytokine levels in serum.

**FIG 4:**
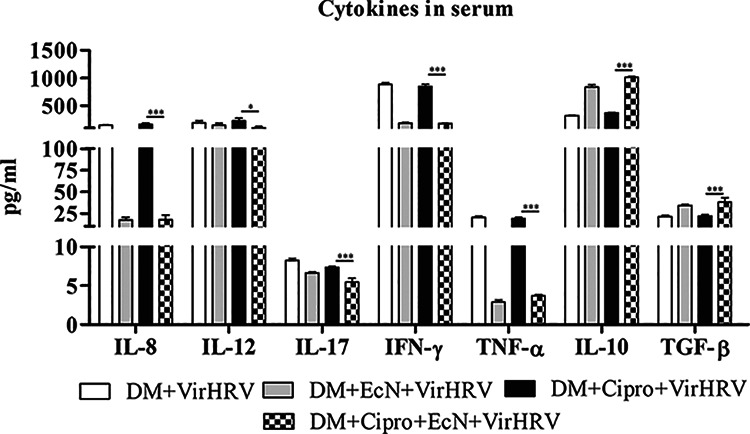
Escherichia coli Nissle 1917 (EcN) treatment modulated proinflammatory and immunoregulatory cytokines in serum from defined commensal microbiota (DM)-transplanted pigs with or without ciprofloxacin (Cipro) after virulent human rotavirus (VirHRV) challenge. Mean concentrations of Th1 (IL-12 and IFN-γ), Th2 (IL-8), Th17 (IL-17), proinflammatory (TNF-α), and T regulatory (IL-10 and TGF-β) cytokines in sera of pigs from different groups are shown. Data are shown as means ± SEM, and significant differences are indicated (*, *P < *0.05; ***, *P < *0.001), as obtained from a nonparametric Kruskal-Wallis rank sum test, for the EcN/Cipro- versus the Cipro-treated groups. Gn neonatal piglets were derived using hysterectomy and transplanted with DM at 7 days of age, followed by challenge with VirHRV 14 days later, and pigs were euthanized at 3 weeks postchallenge (PCD21).

Proinflammatory and immunoregulatory cytokine responses associated with Cipro, EcN, and VirHRV challenge were assessed by measuring the levels of serum cytokines at multiple time points, prechallenge (postchallenge day 0 [PCD0]) and postchallenge (PCD2 and PCD7) (Fig. 4). Coinciding with increased diarrheal scores, Cipro treatment and HRV challenge increased the proinflammatory (interleukin-18 [IL-18] and tumor necrosis factor alpha [TNF-α]) cytokine responses, while EcN treatment reduced the proinflammatory (IL-8, IL-12, IFN-γ, and TNF-α) cytokines at PCD2 (Fig. 4). EcN with or without Cipro treatment reduced IL-17 levels at PCD2 as well as at PCD21 (data not shown). Other cytokines (IL-4, IL-6, and IFN-α) were not altered at the tested time points (data not shown). These data suggest that EcN reduced local (gut) inflammation caused by HRV infection with or without Cipro treatment. In contrast, EcN with or without Cipro treatment increased the immunoregulatory cytokines IL-10 and transforming growth factor β (TGF-β) at PCD2 (Fig. 4). Moreover, the IL-10 cytokine level was increased at PCD0 (data not shown).

### EcN+Cipro treatment decreased the frequencies of TLR4 MNCs and increased the frequencies of TLR3 and TLR9 MNCs.

**FIG 5:**
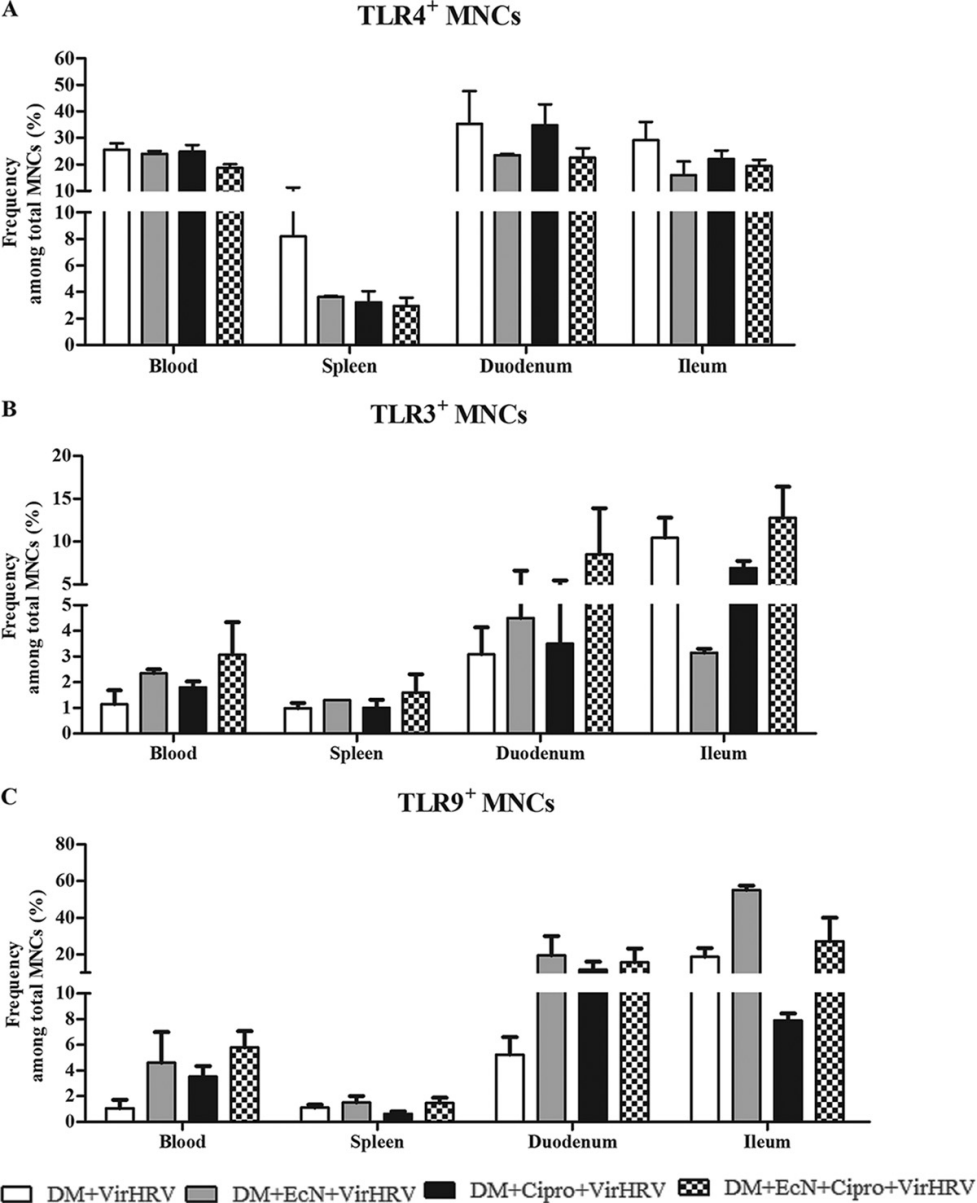
Escherichia coli Nissle 1917 (EcN) treatment modulated the frequencies of Toll-like receptor (TLR)-expressing mononuclear cells (MNCs) in defined commensal microbiota (DM)-transplanted pigs with or without ciprofloxacin (Cipro) after virulent human rotavirus (VirHRV) challenge. Mean frequencies of MNCs expressing TLR4 (A), TLR3 (B), and TLR9 (C) are shown. MNCs were isolated from systemic and intestinal tissues of piglets. Data are shown as means ± SEM. Statistical significance was determined by the nonparametric Kruskal-Wallis test for the EcN/Cipro versus the Cipro groups. Gn neonatal piglets were derived using hysterectomy and transplanted with DM at 7 days of age, followed by challenge with VirHRV 14 days later, and pigs were euthanized at 3 weeks postchallenge (PCD21).

The effects of EcN with and without Cipro treatment on the expression of Toll-like receptor 4 (TLR4), TLR3, and TLR9 were analyzed in systemic and intestinal mononuclear cells (MNCs) (Fig. 5). Coinciding with decreased diarrheal severity, the frequencies of TLR4 (associated with proinflammatory signaling)-expressing MNCs were decreased in systemic and intestinal tissues of EcN-treated pigs (Fig. 5A). The frequencies of TLR3 (associated with anti-RV protection)- and TLR9 (associated with anti-inflammatory signaling)-expressing MNCs were increased in systemic and intestinal tissues of the EcN-treated groups, respectively (Fig. 5B and C). TLR3 and TLR9 expression in ileal tissues was negatively correlated with diarrheal scores (*R* = −0.5 [*P* = 0.04] and *R* = −0.5 [*P *= 0.03], respectively).

### EcN+Cipro treatment increased the frequencies of cDCs, pDCs, activated cDCs and pDCs, and CD103^+^ cDCs/pDCs in systemic and/or intestinal tissues.

**FIG 6:**
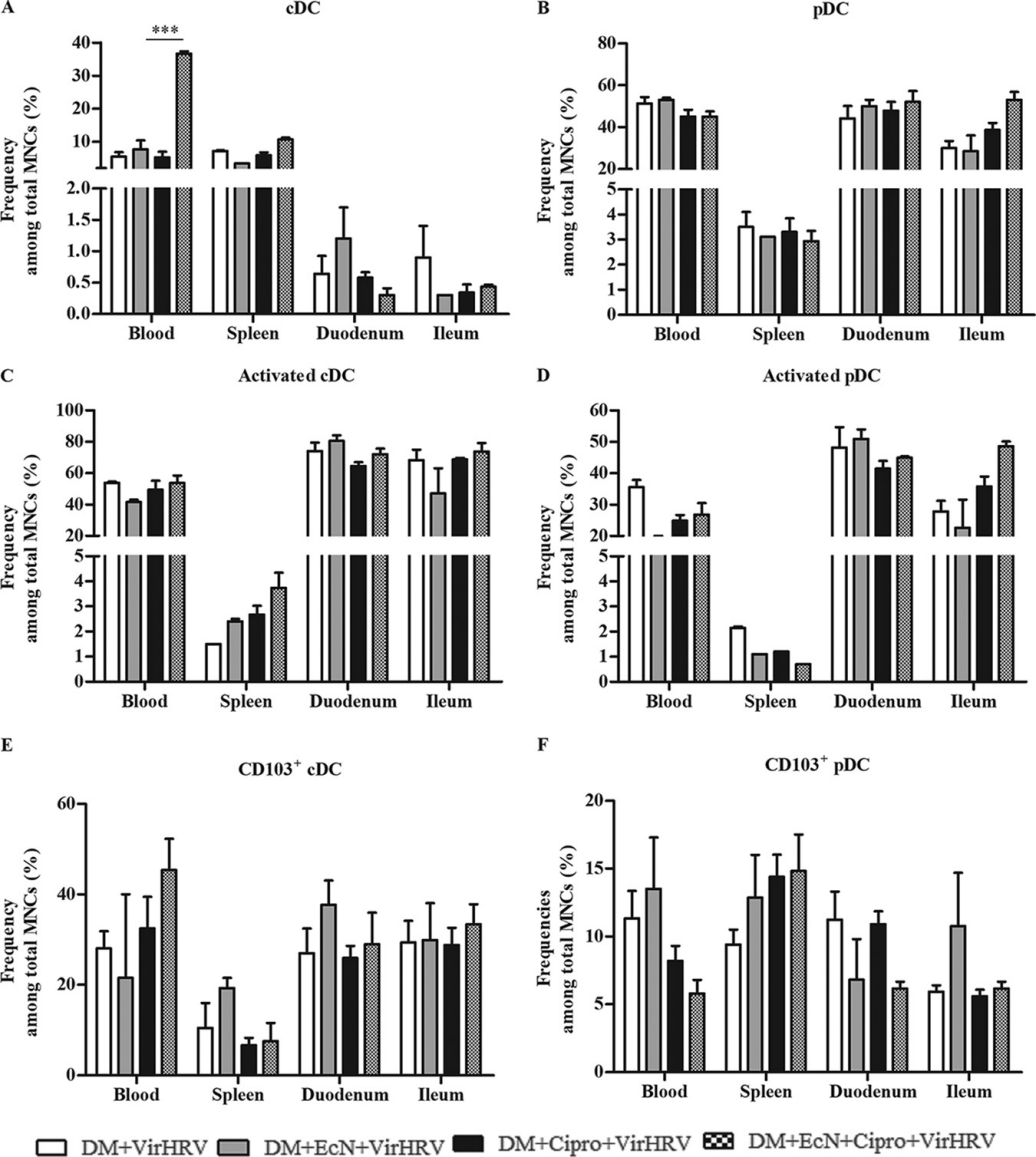
Escherichia coli Nissle 1917 (EcN) treatment alters the frequencies of conventional dendritic cells (cDCs), plasmacytoid dendritic cells (pDCs), activated cDCs and pDCs, CD103^+^ cDCs, and CD103^+^ pDCs in systemic and intestinal tissues in defined commensal microbiota (DM)-transplanted pigs with or without ciprofloxacin (Cipro) after virulent human rotavirus (VirHRV) challenge. Mean frequencies of cDCs (A), pDCs (B), activated cDCs (C), activated pDCs (D), CD103^+^ cDCs (E), and CD103^+^ pDCs (F) are shown. Data are shown as means ± SEM for the EcN/Cipro versus the Cipro groups. Significant differences are indicated (***, *P *< 0.001). Statistical significance was determined by the nonparametric Kruskal-Wallis test. Gn neonatal piglets were derived using hysterectomy and transplanted with DM at 7 days of age, followed by challenge with VirHRV 14 days later, and pigs were euthanized at 3 weeks postchallenge (PCD21). MNCs, mononuclear cells.

EcN treatment with Cipro increased the frequencies of conventional dendritic cells (cDCs) in systemic in blood and ileal tissues (Fig. 6A). The frequencies of cDCs in blood were negatively correlated with diarrheal scores (*R* = −0.5 [*P *= 0.02]). EcN treatment increased the frequencies of plasmacytoid dendritic cells (pDCs) in intestinal tissues (Fig. 6B). Furthermore, EcN treatment with Cipro increased the frequencies of activated cDCs in systemic and intestinal tissues (Fig. 6C) and the frequencies of activated pDCs in blood and intestinal tissues (Fig. 6D). EcN treatment with Cipro increased the frequencies of CD103^+^ cDCs in all tissues (Fig. 6E) and CD103^+^ pDCs in splenic and ileal tissues (Fig. 6F). Moreover, the numbers of CD103^+^ cDCs and pDCs in spleen and duodenal tissues were negatively correlated with diarrheal scores (*R* = −0.5 [*P *= 0.05] and *R* = −0.5 [*P *= 0.02], respectively).

### EcN treatment increased the frequency of NK cells in systemic tissues and NK cell function in blood.

**FIG 7:**
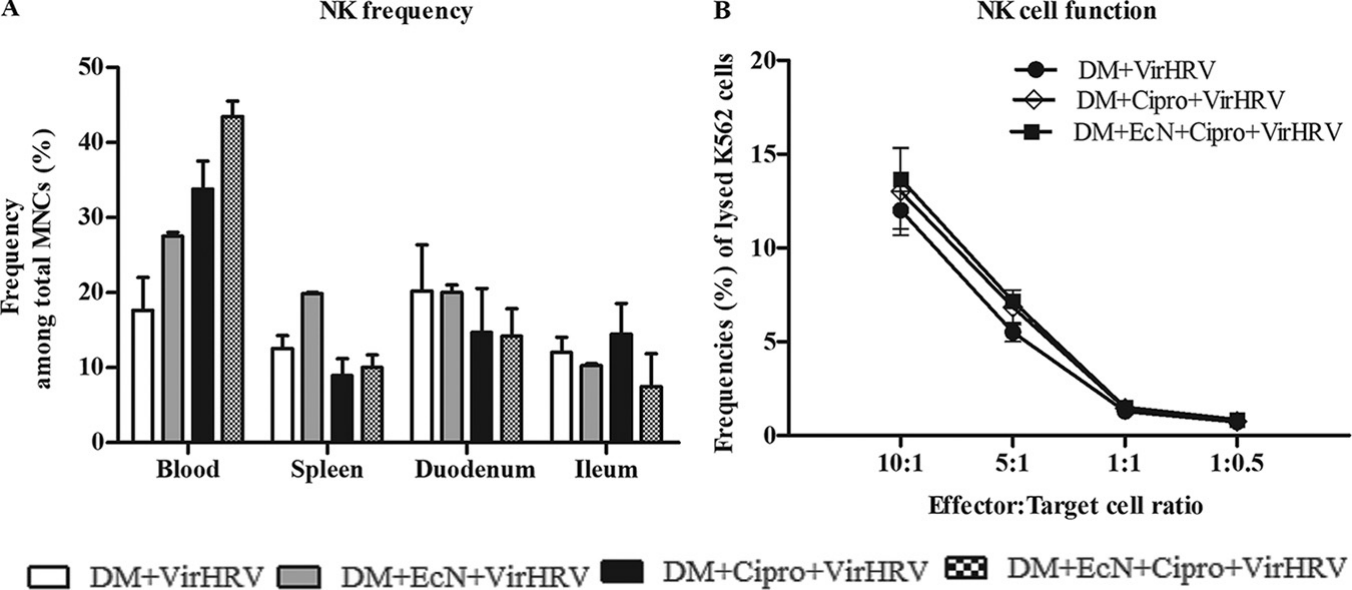
Escherichia coli Nissle 1917 (EcN) enhanced the frequency and function of natural killer (NK) cells in systemic sites in defined commensal microbiota (DM)-transplanted pigs with or without ciprofloxacin (Cipro) after virulent human rotavirus (VirHRV) challenge. The mean frequencies of NK cells (A) and NK cell function (B) in blood mononuclear cells (MNCs) are shown. Blood MNCs and carboxyfluorescein diacetate succinimidyl ester (CFSE)-stained K562 tumor cells were used as effector and target cells, respectively, and cocultured at set ratios to assess NK cytotoxic function. Data are shown as means ± SEM. Statistical significance was determined by the nonparametric Kruskal-Wallis test for the EcN/Cipro versus the Cipro groups. The effector-target cell cocultures were stained with 7-aminoactinomycin D (7AAD) after 12 h of incubation at 37°C, and the frequencies of CFSE-7AAD double-positive cells (lysed K562 target cells) were assessed by flow cytometry. Gn neonatal piglets were derived using hysterectomy and transplanted with DM at 7 days of age, followed by challenge with VirHRV 14 days later, and pigs were euthanized at 3 weeks postchallenge (PCD21).

EcN with or without Cipro treatment increased the frequency of natural killer (NK) cells in systemic tissues (Fig. 7A) and marginally enhanced NK cell cytotoxicity of blood MNCs (Fig. 7B). These data suggest that EcN treatment enhanced innate immune responses associated with Cipro and VirHRV in the Gn pig model.

## DISCUSSION

The human gastrointestinal microbiota and its symbiotic relationship with beneficial microbes play a vital role in immune regulation, including nutrition, metabolism, and pathogen resistance (31–36). However, antibiotics could cause microbial imbalance related to the composition or population of the gastrointestinal microbiota that further compromises mucosal immunity. Microbial imbalance has been associated with health-related problems, including metabolic, immunological, and inflammatory bowel diseases; respiratory diseases such as asthma and allergies; developmental disorders; and increased vulnerability to infectious diseases (37–44). Ciprofloxacin is a folate antagonist broad-spectrum antibiotic that we used for this study. Paim et al. reported that Cipro treatment increased the severity of VirHRV diarrhea in this DM-Gn pig model (20). Moreover, in healthy humans and our DM-Gn pig model, Cipro treatment decreased the taxonomic richness, diversity, and consistency of gut microbiota parameters (26, 45).

Using a DM-transplanted Gn pig model and following treatment with Cipro concurrently with EcN followed by challenge with VirHRV, EcN enhanced multiple aspects of the immune response. Our study demonstrated that EcN treatment enhanced total IgA, IgG, and IgM IgSCs as well as HRV-specific IgA, IgG, and IgM ASCs in systemic and intestinal sites, which coincided with reduced diarrheal scores (20). These results indicate that EcN treatment enhanced HRV antibody-producing cell frequencies and antibody titers in Cipro-treated, VirHRV-challenged, DM-transplanted Gn pigs. This is in agreement with previous studies showing that oral administration of two strains of *Lactobacillus* probiotics increased the number of IgA-positive (IgA^+^) B cells in the lamina propria (46, 47). IgA antibody is a major functional component of the humoral adaptive immune system, especially at mucosal sites (48). The levels of HRV-specific IgA antibodies in pigs strongly correlate with protection against HRV infection (23, 49, 50). Our results confirm that an EcN probiotic enhances the IgA antibody responses. EcN treatment enhanced total and HRV-specific IgA, IgG, and IgM antibody titers in serum, SIC, and LIC. It is possible that the observed effects of EcN treatment on systemic and intestinal IgA responses could be mediated by direct modulation of host immune responses, suggesting that EcN is more stable and persistent in the gut of the host’s gastrointestinal system. Additionally, total and HRV-specific IgM and IgG ASCs and antibody titers were enhanced, further confirming the enhancement of immune responses against HRV infection (see Table S1 in the supplemental material).

10.1128/mSphere.00074-21.5 TABLE S1 Correlation of HRV-specific antibody titers and antibody-secreting cells. Download Table S1, TIF file, 0.1 MB. Copyright © 2021 Michael et al. 2021 Michael et al. https://creativecommons.org/licenses/by/4.0/ This content is distributed under the terms of the Creative Commons Attribution 4.0 International license.

EcN treatment increased the frequencies of activated antibody-forming B cells in systemic and ileal tissues, Ig-secreting B cells in systemic tissues and duodenum cells, resting/memory antibody-forming B cells in intestinal cells, and naive antibody-forming B cells in duodenal cells only. These results are similar to those of our previous studies where EcN protected against HRV infection (6, 51). The frequencies of activated antibody-forming B cells and IgSCs were increased in systemic and ileal cells of EcN-treated pigs, suggesting that EcN potentiated the effect of intestinal B cell development and thus also increased systemic responses. These findings suggest that EcN treatment enhanced B cell immune responses in systemic tissues, with some effects on intestinal tissues. These responses coincided with reduced diarrhea (20) and increased HRV-specific IgA antibody responses in serum, SIC, and LIC.

Innate immune responses are critical as the first line of defense, limiting RV replication and disease severity in the host (18, 52) as well as shaping humoral immune responses. EcN treatment enhanced innate immune responses. For example, NK cell frequencies and cytotoxicity were marginally increased in systemic sites of mice in the EcN-treated groups. This suggests that EcN treatment promoted innate immune responses, improving protection against HRV infection *in vivo.* This suggests that EcN administration can inhibit the proapoptotic effects of HRV infection by (i) inhibiting proinflammatory TLR-mediated proapoptotic signaling or activating antiapoptotic pathways (18) and (ii) supporting adequate immune function and programmed cell death (53, 54).

Dendritic cells (DCs) play a key role in the interaction with probiotic bacteria and initiation of the innate immune responses (55, 56), and pDCs were shown to contribute to RV clearance in a murine model (57). Moreover, DC major histocompatibility complex class II (MHC-II) expression is a marker of maturation (58). In our study, EcN treatment with Cipro increased the frequencies of CD103^+^ cDCs in all tissues, CD103^+^ pDCs in the spleen, cDCs in the ileum and systemic tissues, pDCs in intestinal tissues, and activated cDCs and activated pDCs in systemic and/or intestinal tissues. These results suggest that EcN was stable in the gut and thus enhanced the maturation of systemic and intestinal activated DCs, promoted pDC and cDC development, and increased IgA antibody responses in Cipro- and VirHRV-treated pigs (59, 60). Enhancing the induction of pDCs with EcN may be critical in protection against enteric pathogens (18). Moreover, the enhanced activation of B cells coincided with increased frequencies of cDCs and pDCs (51). Also, lamina propria DCs expressing CD103 that were enhanced in our study are known to switch naive CD4^+^ T cells into FOXP3^+^ T regulatory cells (61). In this study, we observed reduced CD103^+^ cDCs in systemic and intestinal tissues in Cipro- and VirHRV-treated pigs. The loss of CD103 (α_E_β_7_) integrin by intestinal DCs during experimentally induced colitis was investigated in mice (62), suggesting that Cipro/VirHRV-associated MNC necrosis, possibly showing intestinal inflammation in our DM-Gn pig model, may have resulted in reduced CD103^+^ DC frequencies. Interestingly, EcN treatment increased the expression of CD103^+^ DCs in systemic and intestinal tissues, probably by reducing the number of necrotic MNCs. Moreover, CD103^+^ DCs are implicated in maintaining tight junction proteins, protecting the integrity of the epithelial barrier, and preventing inflammatory reactions to intestinal pathogens, and they affect cellular intraepithelial motility and morphogenesis (63, 64). Additionally, CD103 integrin is essential for proper communication between the pathogen, DCs, and T/B lymphocytes (65). Therefore, the Cipro/VirHRV-induced decreased frequencies of CD103-expressing DCs that we observed could have resulted in atypical innate immune signaling against Cipro/VirHRV and worsening of the infection. On the other hand, EcN treatment enhanced HRV-specific IgA ASCs and antibody titers, improved the epithelial barrier, and reduced diarrhea severity (20).

Previous studies have demonstrated that TLR2, -4, -7, and -8 expression in peripheral blood MNCs of pediatric patients is upregulated during HRV infection (66). TLR4 expressed by epithelial and immune cells plays an important role in the mucosal host defense against invading pathogens. Moreover, probiotic bacteria downregulated TLR4 expression associated with proinflammatory and proapoptotic signaling (54, 67–70). Consistent with previous observations (18, 51), we demonstrated that HRV-induced TLR4-expressing MNC frequencies were reduced in systemic and intestinal MNCs by EcN treatment. TLR3 is involved in the initial recognition of RV genomic double-stranded RNA (dsRNA). In this study, EcN treatment increased the TLR3^+^ MNC frequencies in systemic and intestinal MNCs, suggesting that EcN probiotic colonization may have supported immune activation of virus-induced TLR3^+^ MNCs or enhanced their persistence. TLR3-mediated immune responses are associated with limiting RV replication (51, 71). TLR9^+^ MNC frequencies were increased in the systemic and intestinal MNCs of EcN-treated pigs, coinciding with increased protection against diarrhea (20). This suggests a potent beneficial effect of EcN leading to the upregulation of TLR9 expression in systemic and intestinal MNCs. Thus, increased TLR9 expression in EcN-treated pigs could contribute to the enhanced immunoglobulin responses observed (6, 72, 73). Moreover, these findings are consistent with previous findings that anti-inflammatory signaling via TLR9 resulted in decreased ulcerative colitis and Helicobacter pylori-induced gastritis in mice (74, 75). These results indicate that enhanced TLR3/TLR9 expression facilitated more efficient recognition of RV and RV dsRNA, improving protection against HRV diarrhea. Moreover, they suggest that EcN treatment, by upregulating TLR9 expression, could result in enhanced antibodies against HRV and the increased proinflammatory responses observed.

EcN treatment enhanced T cell immune responses by increasing the frequencies of CD3^+^ CD4^+^ T cells in the blood and duodenum (postchallenge), CD3^+^ CD8^+^ T cells in the spleen and intestinal tissues (pre/postchallenge), and splenic and ileal CD3^+^ CD4^+^ and CD3^+^ CD8^+^ IFN-γ-producing T cells (postchallenge). The latter coincided with decreased splenic CD4^+^ Tregs (postchallenge) and splenic and ileal CD3^+^ CD8^+^ Tregs (postchallenge). This suggests that EcN modulates the immunoregulatory environment with or without Cipro treatment, serves as a potent inducer of intestinal immunity, restores gut homeostasis, and thus moderates HRV infection and Cipro treatment effects post-VirHRV challenge. The frequencies of T helper or T cytotoxic cells in blood were correlated with reduced virus shedding (20). IFN-γ-producing T cells have previously been correlated with protection against HRV infection in pigs (76, 77).

The higher serum levels of the immunoregulatory cytokines TGF-β and IL-10 might have contributed to the reduced serum levels of the proinflammatory cytokines IL-8, TNF-α, IL-17, and IL-12 associated with EcN treatment. Induction of an anti-inflammatory microenvironment may have reduced HRV-induced disease (20) or the subsequent aggravated host immune responses (78–80). We observed higher levels of the Th1 cytokines IFN-γ and IL-12 in Cipro-treated HRV-challenged pigs, while reduced levels were detected in pigs treated with EcN with or without Cipro, suggesting a Th1-induced microenvironment during HRV infection that coincided with higher diarrheal severity scores (20). We observed higher anti-inflammatory IL-10 levels in EcN-treated pigs, which may also contribute to the higher HRV IgA antibody responses (81) observed in these pigs, possibly through TLR9 signaling (82). IL-17 is a proinflammatory cytokine and plays a critical role in host defense and inflammatory and autoimmune diseases (83). We observed higher serum IL-17 levels in the groups treated with VirHRV with or without Cipro than in the EcN-treated groups. Similar findings were observed previously for influenza virus and respiratory syncytial virus infections (84, 85). Consistent with our previous observations using probiotics (5) and in EcN-treated pigs, lower IL-17 levels were observed, thus indicating an ameliorated HRV inflammatory response. This suggests that EcN induced an anti-inflammatory environment with or without Cipro treatment post-VirHRV challenge, thereby inhibiting proinflammatory cytokine responses.

In summary, our results suggest that Cipro treatment may have perturbed gastrointestinal homeostasis, which resulted in altered immune responses, whereas the probiotic EcN promoted strong but balanced immunoregulatory/immunostimulatory responses during VirHRV infection of DM-colonized Gn piglets. Our results suggest that low-cost dietary supplementation with EcN can protect against antibiotic-associated diarrhea and potentially other enteric infections. Further studies are necessary to investigate the EcN efficacy under conditions where children are exposed to antibiotics and malnutrition and in Gn pigs colonized with a complete human infant microbiota.

## MATERIALS AND METHODS

### Virus.

The virulent HRV (VirHRV) Wa strain passaged 25 to 26 times in Gn piglets was used to orally inoculate piglets at a dose of 2 × 10^6^ fluorescent focus units (FFU) as described previously (86, 87).

### Animal experiments.

This study was approved by The Ohio State University Institutional Animal Care and Use Committee. Piglets were derived from near-term sows (Landrace × Yorkshire × Duroc cross-bred) by hysterectomy and maintained in sterile isolators as described previously (88). All piglets were colonized orally at 7 days of age with defined commensal microbiota (DM) with 10^5^ CFU of each bacterium/piglet (26). DM were kindly provided by David Francis from South Dakota State University. The experimental design was adapted from that previously described (20), wherein piglets were randomly assigned to 4 groups (Fig. 1A): DM+VirHRV (*n* = 7), DM+Cipro+VirHRV (*n* = 6), DM+EcN+VirHRV (*n* = 3), and DM+Cipro+EcN+VirHRV (*n* = 4). The piglets were orally treated or untreated with Cipro (60 mg/day) and/or EcN (10^5^ CFU/piglet daily) at post-bacterial transplantation day 8 (PBTD8) to PBTD13. The EcN inoculum was prepared as described previously (19). All piglets were challenged with VirHRV at a dose of 2 × 10^6^ FFU per piglet at PBTD14 and euthanized by electrocution following anesthesia at PBTD35/post-VirHRV challenge day 21 (PCD21). Non-DM Gn and conventional piglets were not included because their inclusion would significantly complicate this already complex experimental design. The primary goal of this study was to evaluate the effects of Cipro and EcN treatments in a microbiota-associated pig model without multiple compounding factors characteristic of studies in conventional animals. One of the variables found in conventional pigs that would be a significant compounding factor is the natural variability of the gut microbiome. The non-DM piglets that were included in our previous experiments generally demonstrate the same trends, but the effects are less pronounced. The blood, spleen, duodenum, and ileum were collected to isolate mononuclear cells (MNCs) for subsequent immunological assays. Serum, small intestinal contents (SIC), and large intestinal contents (LIC) were collected to determine the HRV-specific and total antibody responses (6, 19, 86, 89, 90).

### Isolation of mononuclear cells.

Systemic (blood and spleen) and intestinal (duodenum and ileum) tissues were collected to isolate MNCs as described previously (5, 23, 76, 91, 92). The purified MNCs were suspended in E-RPMI 1640. The viability of each MNC preparation was determined by trypan blue exclusion (≥95%).

### HRV-specific and total antibody responses.

The HRV antibody and total immunoglobulin (Ig) isotype titers in serum, SIC, and LIC were detected by an enzyme-linked immunosorbent assay (ELISA) as described previously (6, 19, 86, 89, 90, 92). To determine the intestinal antibody responses, SIC and LIC were collected with protease inhibitors in the medium.

### HRV-specific antibody-secreting cell and total Ig-secreting cell responses.

HRV-specific antibody secretion in MNCs isolated from the blood, spleen, duodenum, and ileum was analyzed by an enzyme-linked immunosorbent spot (ELISPOT) assay as described previously (6, 19, 89, 90, 92).

### Serum cytokines.

Serum samples were collected at multiple time points and analyzed for proinflammatory (TNF-α and IL-6), innate (IFN-α), Th1 (IL-12 and IFN-γ), Th2 (IL-4, IL-6, and IL-8), and Treg (IL-10 and TGF-β) cytokines as described previously, with some modifications (5, 79, 91).

### Flow cytometry analysis.

Freshly isolated MNCs were stained for determining the following T cell subsets: T helper cells (CD3^+^ CD4^+^), cytotoxic T cells (CD3^+^ CD8^+^), and T regulatory cells (CD4^+^/CD8^+^ CD25^+^ FOXP3^+^) (5, 91). To determine the frequencies of HRV-specific IFN-γ-producing CD4^+^ and CD8^+^ cells, freshly isolated MNCs from the spleen and ileum were restimulated *in vitro* with the semipurified attenuated HRV Wa strain (12 μg/ml) and porcine cross-reactive human CD49d monoclonal antibody (mAb) (0.5 μg/ml) (clone 9F10; BD Pharmingen) for 18 h and stained as previously described (5, 91). MNCs were stained to assess the frequencies of conventional dendritic cell (cDC) (SWC3a^+^ CD4^−^ CD11R1^+^), plasmacytoid DC (pDC) (SWC3a^+^ CD4^+^ CD11R1^−^), activated cDC (SWC3a^+^ CD4^−^ CD11R1^+^ MHC-II^+^), activated pDC (SWC3a^+^ CD4^+^ CD11R1^−^ MHC-II^+^), CD103^+^ cDC (SWC3a^+^ CD4^−^), and CD103^+^ pDC (SWC3a^+^ CD4^+^) marker expression on DCs and Toll-like receptor (TLR) expression on MNCs with monoclonal antibodies to porcine and human cell surface markers as reported previously (6, 18, 54, 93). TLR3 (ligand double-stranded RNAs), TLR4 (ligand bacterial lipopolysaccharide), and TLR9 (ligand bacterial CpGs) were used in our experiments. Similarly, the frequencies of resting/memory antibody-forming B cells (CD79β^+^ CD2^−^ CD21^−^), Ig-secreting B cells (CD79β^+^ CD2^−^ CD21^+^), naive antibody-forming B cells (CD79β^+^ CD2^+^ CD21^+^), and activated antibody-forming B cells (CD79β^+^ CD2^+^ CD21^−^) among systemic and intestinal CD79β^+^ B cells were determined as described previously (6, 19, 94). The frequencies of NK cells (SWC3a^+^ CD16^+^) were assessed among systemic and intestinal MNCs. Appropriate isotype-matched control antibodies were included. Subsequently, 50,000 events were acquired per sample using a BD Accuri C6 flow cytometer (BD Biosciences, San Jose, CA, USA). Data were analyzed using C6 flow sampler software.

### NK cytotoxicity assay.

Total blood MNCs and K562 cells were used as effector and target cells, respectively. Effector-to-target cell ratios of 10:1, 5:1, 1:1, and 0.5:1 were used, and the assay was done as described previously (91, 95).

### Statistical analysis.

All statistical analyses were performed using GraphPad Prism version 6 (GraphPad Software, Inc., La Jolla, CA). Log_10_-transformed isotype ELISA antibody titers were analyzed using one-way analysis of variance (ANOVA) followed by Duncan’s multiple-range test. Correlation analysis was performed using Spearman’s nonparametric correlation method. Data represent the mean numbers of HRV-specific antibody-secreting cells per 5 × 10^5^ MNCs and were analyzed using a nonparametric *t* test (Mann-Whitney) (*, *P* value of <0.05; **, *P* value of <0.01; ***, *P* value of <0.001). Error bars indicate the standard errors of the means (SEM).
