# Supplementary figures and images for: Escherichia coli Nissle 1917 Enhances Innate and Adaptive Immune Responses in a Ciprofloxacin-Treated Defined-Microbiota Piglet Model of Human Rotavirus Infection
Source: mSphere. 2021 Mar 31;6(2):e00074-21. doi: 10.1128/mSphere.00074-21 (PMC8546683; doi:10.1128/mSphere.00074-21)

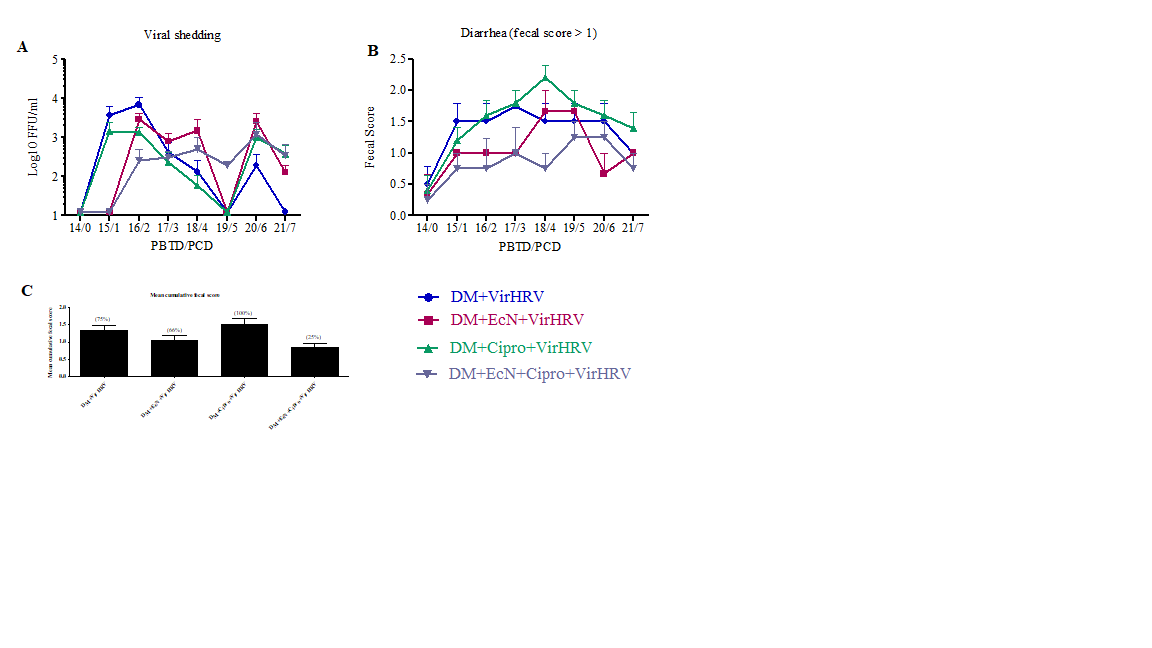

Supplement: FIG S1 [file msphere.00074-21-sf001.tif]

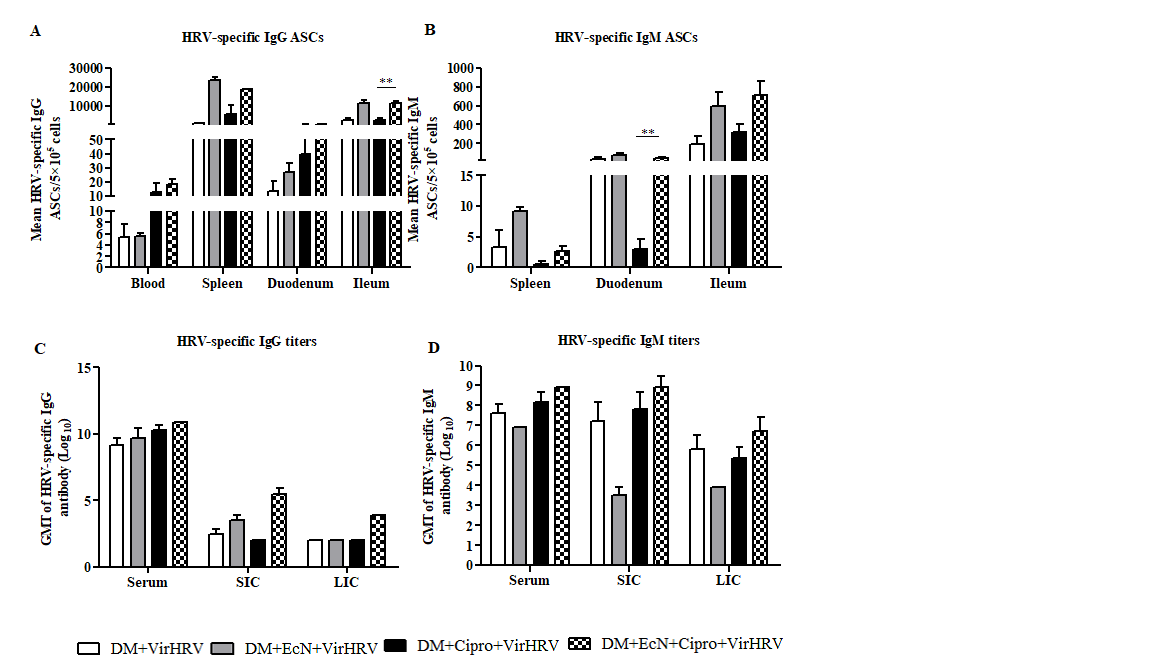

Supplement: FIG S2 [file msphere.00074-21-sf002.tif]

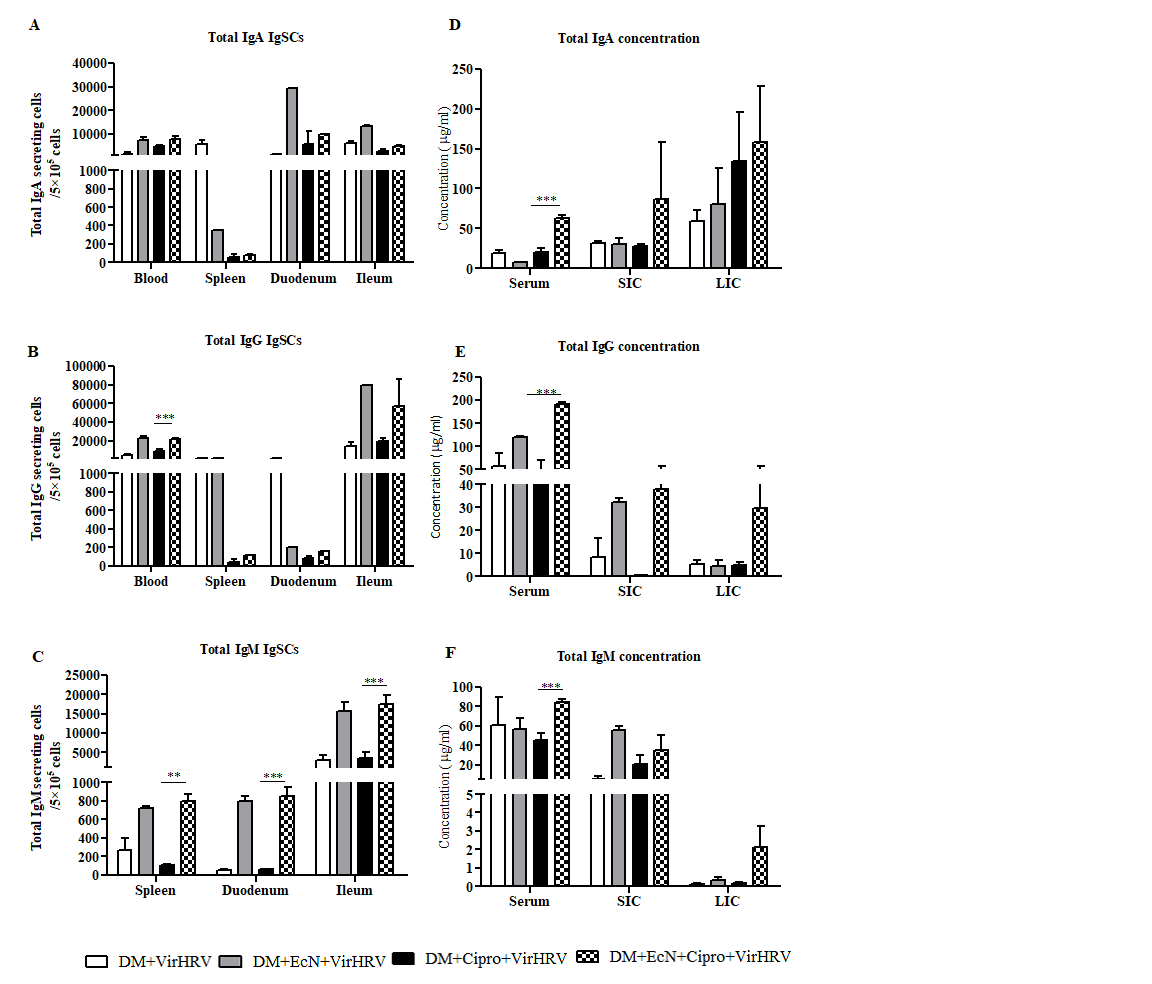

Supplement: FIG S3 [file msphere.00074-21-sf003.tif]

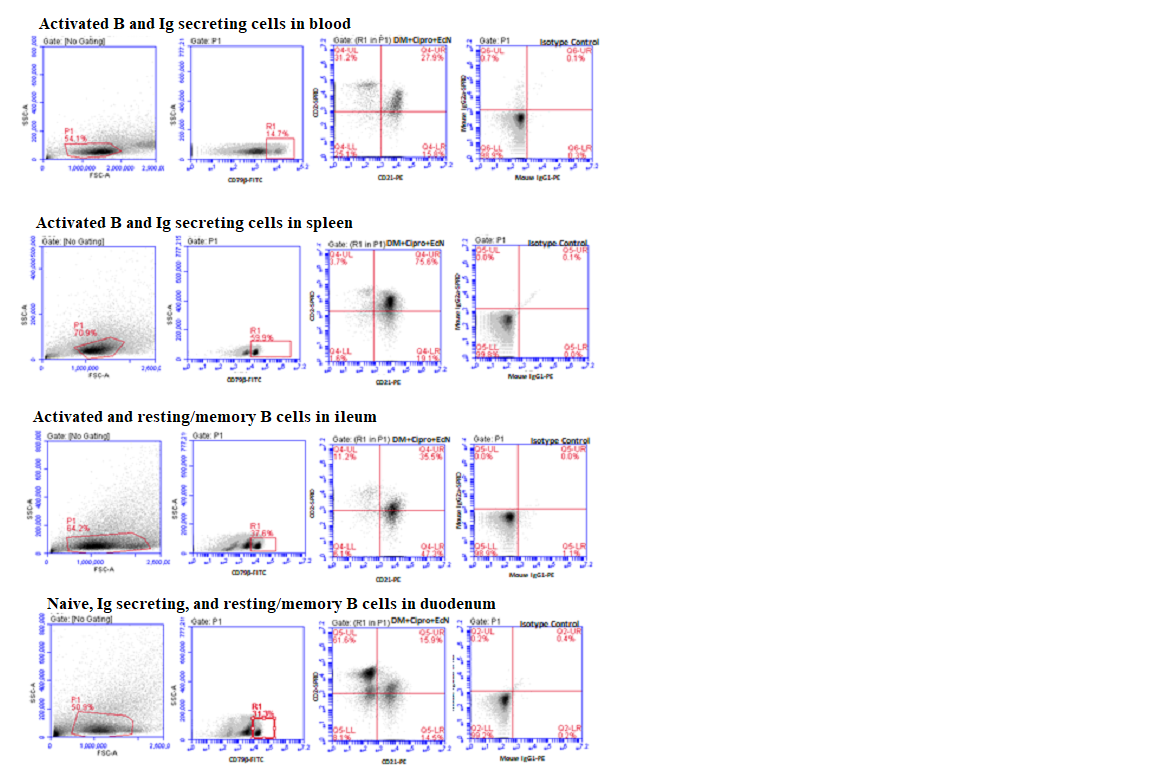

Supplement: FIG S4 [file msphere.00074-21-sf004.tif]

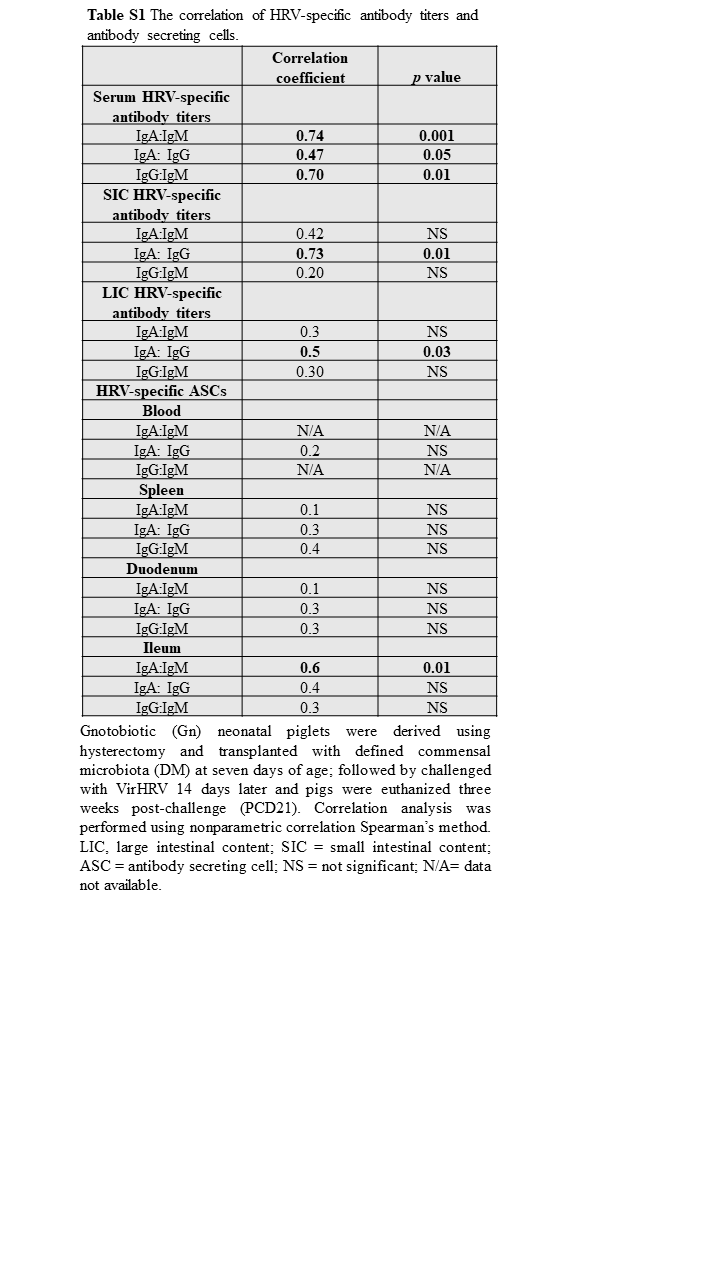

Supplement: TABLE S1 [file msphere.00074-21-st001.tif]
